# Comparative Study on Trace Element Excretions between Nonanuric and Anuric Patients Undergoing Continuous Ambulatory Peritoneal Dialysis

**DOI:** 10.3390/nu8120826

**Published:** 2016-12-20

**Authors:** Siyun Xiang, Ying Yao, Yunan Wan, Wangqun Liang, Ruiwei Meng, Qiman Jin, Nannan Wu, Fangyi Xu, Chenjiang Ying, Xuezhi Zuo

**Affiliations:** 1 Department of Clinical Nutrition, Tongji Hospital, Tongji Medical College, Huazhong University of Science and Technology, Wuhan 430030, Hubei, China; sy_xiang@hust.edu.cn (S.X.); yaoyingkk@126.com (Y.Y.); 2 Department of Nutrition and Food Hygiene, School of Public Health, Tongji Medical College, Huazhong University of Science and Technology, Wuhan 430030, Hubei, China; wanyunan1122@163.com (Y.W.); JinQiMan1994@163.com (Q.J.); m15927511308@163.com (N.W.); xufangyi4527@126.com (F.X.); 3 Department of Nephrology, Tongji Hospital, Huazhong University of Science and Technology, Wuhan 430030, Hubei, China; lwq19710228@163.com; 4 Department of Epidemiology and Biostatistics, School of Public Health, Tongji Medical College, Huazhong University of Science and Technology, Wuhan 430030, Hubei, China; meng_ruiwei@163.com

**Keywords:** trace element, continuous ambulatory peritoneal dialysis, excretion, selenium, molybdenum

## Abstract

Few studies have been reported on alterations of trace elements (TE) in peritoneal dialysis patients. Our objective was to investigate and assess the characteristics of daily TE excretions in continuous ambulatory peritoneal dialysis (CAPD) patients. This cross-sectional study included 61 CAPD patients (nonanuric/anuric: 45/16) and 11 healthy subjects in Wuhan, China between 2013 and 2014. The dialysate and urine of patients and urine of healthy subjects were collected. The concentrations of copper (Cu), zinc (Zn), selenium (Se), molybdenum (Mo), and arsenic (As) in dialysate and urine were determined using inductively coupled plasma mass spectrometer (ICP-MS). Various clinical variables were obtained from automatic biochemical analyzer. Daily Cu, Zn, Se, and Mo excretions in nonanuric patients were higher than healthy subjects, while arsenic excretion in anuric patients was lower. A strong and positive correlation was observed between Se and Mo excretion in both dialysate (β = 0.869, *p* < 0.010) and urine (β = 0.968, *p* < 0.010). Furthermore, the clinical variables associated with Se excretion were found to be correlated with Mo excretion. Our findings indicated that nonanuric CAPD patients may suffer from deficiency of some essential TEs, while anuric patients are at risk of arsenic accumulation. A close association between Se and Mo excretion was also found.

## 1. Introduction

Over the last 10 years, chronic kidney disease (CKD) has received increased attention as a leading public health problem. The incidence and prevalence of CKD are increasing rapidly worldwide due to the speedy increase in the prevalence of risk factors such as diabetes, hypertension, and obesity. These factors will also foreseeably result in a greater burden of CKD in the future [1,2]. In China, the overall prevalence of CKD adults is 10.8% [3,4], which may have substantial socioeconomic and public health consequences. It has long been recognized that CKD patients suffer from disturbance of macronutrients, such as protein-energy malnutrition and disorders of calcium-phosphate metabolism [5,6,7]. However, a few studies have investigated the status of trace elements (TEs).

Some vital biochemical functions of humans depend significantly on TEs like copper (Cu), zinc (Zn), selenium (Se), and molybdenum (Mo). These elements play an important role in human health and are recognized by the U.S. Department of Agriculture (USDA) as essential TEs [8]. Other TEs like arsenic (As) and lead (Pb) are potentially harmful to humans [8].

Because many TEs are eliminated by the kidney, their metabolisms may become complex for patients with CKD requiring continuous ambulatory peritoneal dialysis (CAPD). In these patients, according to the principle of osmotic ultrafiltration, some TEs may be removed, whereas others could be transferred into patients with the peritoneum serving as the selective semipermeable membrane [9]. As a result, TEs in lower concentrations in the dialysis fluids may be leached from the body. Especially, patients are at risk for low dietary intake of such substances which is attributable to uremia-related anorexia and dietary restrictions. Based on these, some essential TEs may be deficient, thereby causing some complications, which may further lead to an acceleration in disease progression [10,11,12,13,14]. On the other hand, toxic TEs that present in dialysis fluids but not in blood may accumulate. Therefore, TE disturbances might occur in this population.

Alterations in the metabolism of Cu, Zn, and Se have been frequently observed in patients with chronic kidney disease (CKD) [15,16,17,18]. Anorexia, low taste sensibility, glucose intolerance, healing difficulties, anemia, malnutrition, and cardiovascular disease are common features and complications of CKD that can be associated with these alterations [10,11,12,13,14]. The positive effects of these element supplements on CKD patients are also supported by some research studies [19,20]. The majority of the studies that evaluated the status of these TEs focused on CKD patients without dialysis treatment and those receiving hemodialysis (HD), while limited investigations have been conducted regarding CAPD patients [9,21,22,23,24,25,26]. In addition, few studies have been reported on the status of TE excretions in CAPD patients under the special circumstance of this disease, which would provide suggestions for clinical prevention and treatment and would even be available to establish recommendations for supplementation of deficient TEs. Thus, further studies on CAPD patients are required.

Urine volume reflects the residual renal function of CAPD patients. With regard to anuric patients defined as 24-h urine volume <100 mL [27] on PD, the excretions mainly depend on dialysis treatment, which makes a difference. Therefore, the aim of the present study is to investigate the excretions of daily Cu, Zn, Se, Mo, and As and assessing their characteristics in CAPD patients, including the differences of TEs excretions between anuric patients (urine output < 100 mL/day) and a nonanuric group (urine output > 100 mL/day).

## 2. Materials and Methods

### 2.1. Study Participants

Between January 2013 to October 2014, 65 CKD patients receiving CAPD (34 males, 31 females) were recruited in Tongji Hospital, a teaching hospital affiliated to Huazhong University of Science & Technology, Wuhan, China. The inclusion criteria were as follows: (1) non-diabetic nephropathy patients; (2) aged more than 18 years; and (3) treated with CAPD for longer than three months. Patients who had a recent episode of peritonitis or other infectious complications 30 days prior to the sample collection were excluded. Eleven healthy subjects (six males, five females) were taken as a reference. The study protocol was approved by the Hospital Ethics Committee (IRB ID: TJ-C20120501) and all the subjects signed the informed consent form.

### 2.2. Demographic and Anthropometric Data

Demographic parameters and anthropometric measurements—such as gender, age, height, and body weight—were collected from the electronic medical records. The duration of dialysis (month) was calculated as the interval between the date of initiation of dialysis and the date of the sample collection.

### 2.3. Dialysate Information

The applied dialysate was manufactured by Baxter Healthcare and contained 1.5% glucose and 2.5% glucose, respectively. The dosage varied from 4 to 10 L each day and 2 L per bag of standard hypertonic dialysate fluid was infused into the abdominal cavity and remained for 4 h.

### 2.4. Biochemical Measurements

All serum biochemistry parameters including total protein, albumin, fasting blood glucose, creatinine, uric acid, and urea nitrogen were measured. Weekly total urea clearance (Kt/V) and weekly total creatinine clearance (Ccr) were calculated from a 24-h collection of dialysate and urine with the use of standard methods [28]. The residual glomerular filtration rate (eGFR) was calculated as an average of the 24-h urinary urea and creatinine clearances. All biochemistry measurements were performed with an auto-biochemistry analyzer machine at the central laboratory of Tongji Hospital.

Parameters in 24-h urine and spent dialysates (effluent) including the volume, urea nitrogen, total protein, albumin, and creatinine were determined. The normalized protein nitrogen appearance (nPNA) and normalized protein catabolic rate (nPCR) were calculated with the formula by Teehan et al. [29] and Kopple et al. [30]. All measurements were carried out in accordance with the national criteria.

### 2.5. Sample Collection, Storage, and Determination of TEs

We collected 24-h urine from both CAPD patients and healthy subjects. The 24-h spent dialysate was collected from CAPD patients only. Different batches of fresh dialysate were also collected. All samples were collected into cleaned conical 50-mL polypropylene tubes sealed with O-ring screw caps, packed into coolers with frozen ice packs, stored at −80 °C in the laboratory, sequentially and immediately.

The concentrations of TEs including Cu, Zn, Se, Mo, and As in fresh and spent dialysate samples as well as urine samples were measured by an inductively coupled plasma mass spectrometry with an octopole-based collision/reaction cell (Agilent 7700 Series, Waldbronn, Germany) following the method reported by Feng et al. [31]. Accuracy and precision of the results were guaranteed by the analysis of standard reference materials (SRMs) 2670a and 1640a. The two SRMs were purchased from NIST (National Institute of Standards and Technology, Gaithersburg, MD, USA). The relative standard deviation (RSD) of the duplicate analyses (three times) for the five metals in each urine sample was calculated to assess the accuracy. The concentration of the metal was re-quantified if RSD was greater than 10%. In addition, we estimated the determination accuracy of these metals by comparing the difference between the certified values available and the measured values with their uncertainty. The measurement results by our method were in agreement with the SRM 2670a certified values. At the same time, SRM1640a was always analyzed after every 20 samples to ensure instrument performance, which was certified for all metals. Copper, zinc, and arsenic in SRM 2670a were not certified; nonetheless, the NIST provided reference or information values. The mean results of copper, zinc, and arsenic by the method agreed within 10.7% of the target value. The instrument was recalibrated using multi-element standards and the previous 20 samples were reanalyzed if their concentrations were significantly different from actual concentrations. The limits of quantification (LOQ) for the metals were in the range 0.0010–0.2336 μg/L (Shown in Table A1). The concentrations of samples below the LOQ were replaced with LOQ/2.

### 2.6. Statistical Analysis

Data were presented as mean ± standard deviation, median, and range as appropriate for continuous variables, or percentages (%) for categorical variables. EpiData 3.0 (The EpiData Association, Odense, Denmark) was used for the double-key data entry and the program control of the data entry. The statistical analysis was performed with SPSS 18.0 (SPSS Inc., Chicago, IL, USA) for WINDOWS. To identify differences between or among groups, data were analyzed using the Mann-Whitney test or Kruskal-Wallis test. The Wilcoxon matched pairs signed rank was used to assess differences between TEs excretions in dialysate and urine. We used Chi-square test for categorical variables. The association between TEs and other parameters were analyzed by the Spearman rank correlations. Statistical significance was set at *p* < 0.05.

## 3. Results

### 3.1. Study Population

The basic characteristics of the study subjects were summarized in Table 1. The 61 participants in the analyses included 32 males and 29 females. The mean age was 43.31 ± 12.67 years. They had undergone CAPD for 12.52 months (ranging from 3 to 56 months). The mean BMI of CAPD patients was 21.60 kg/m^2^ and 7 patients (11.5%) were with BMI less than 18.5 kg/m^2^ while 10 patients (16.4%) were with BMI greater than 24 kg/m^2^. Out of 61 patients, 45 (73.7%) had the Kt/V over 1.7 and Ccr over 50, which are recommended by the Peritoneal Dialysis Standard Operating Procedure of China, as indices for peritoneal dialysis adequacy.

### 3.2. The Results of Trace Element Excretions

**Figure 1:**
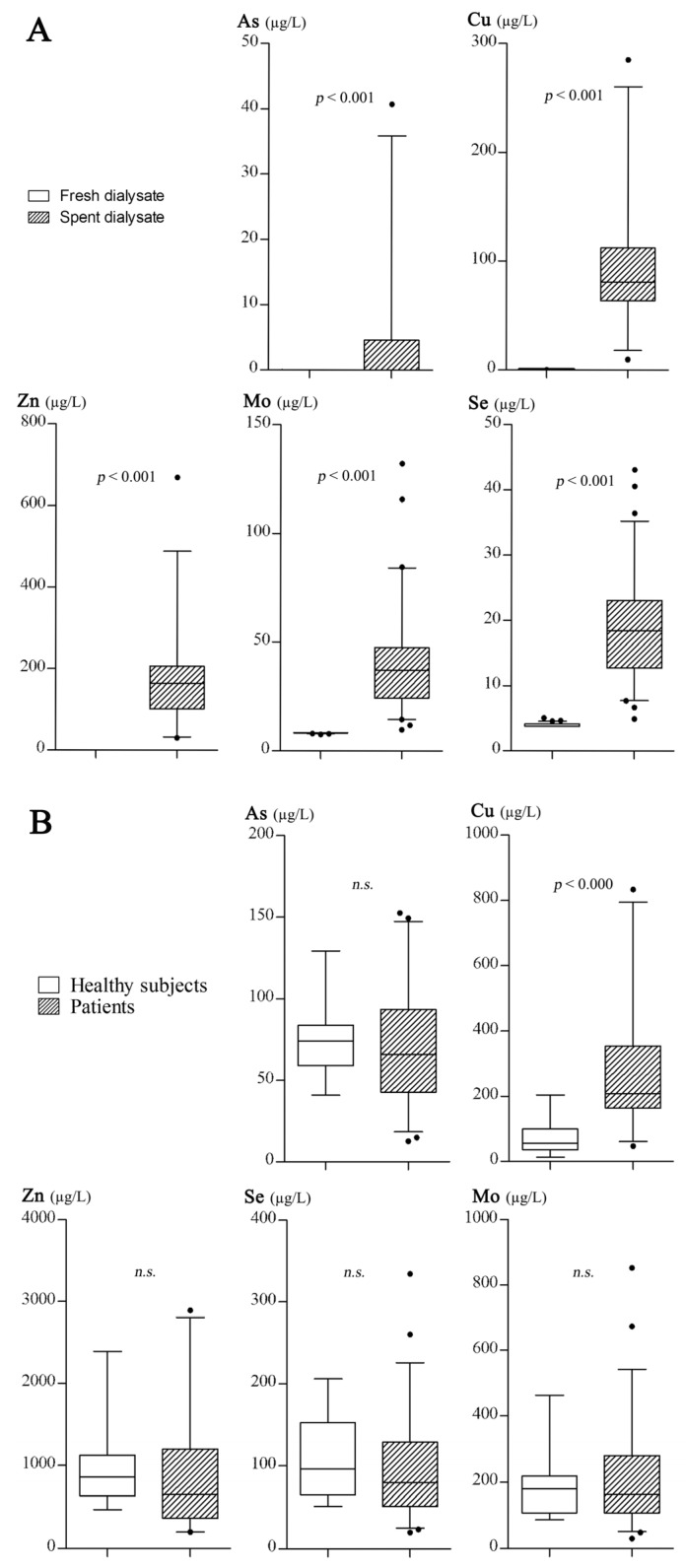
(**A**) The comparison of trace element concentrations between fresh and spent dialysate (effluent); (**B**) The comparison of urinary trace element concentrations between patients and healthy subjects. n.s.: no significant difference.

Figure 1A reveals the comparisons of TEs between fresh and spent dialysate. Figure 1B shows the comparisons of urinary TEs between patients and healthy subjects—including Cu, Zn, Se, Mo, and As. According to the results, the TE concentrations in spent dialysate were found to be significantly higher in comparison with the fresh dialysate. Urinary Cu in patients was higher than the healthy controls.

CAPD patients were divided into two groups according to urine output status: anuric group (*n* = 16) and nonanuric group (*n* = 45). The basic characteristics were shown in Table 2. Compared with healthy participants, CAPD patients were more likely to be older. While, there was no significant difference between them. Anuric patients received longer dialysis treatment when compared with nonanuric patients. It also presents the daily TEs excretions and comparisons of these excretions between patients and healthy subjects. The losses of TEs by dialysate were estimated by comparing the amounts of TEs in spent dialysate with fresh dialysate in 24 h. The losses of TEs in the urine were estimated from their amounts in the collected 24-h urine samples. Besides, total daily losses of TEs in CAPD patients per day were evaluated by the sum of these element amounts excreted by dialysate and urine. There was a significant difference in age of CAPD patients and healthy control subjects (*p* < 0.05) as referred before, then subjects were divided into three age groups: 20–40 years, 40–60 years, and 60–80 years. No significant differences of TEs losses were observed between these three groups. Therefore, healthy control subjects can be taken as a reference. The comparison of daily TEs losses between patients and healthy subjects showed that the daily Cu losses in patients were significantly higher relative to the healthy subjects. Furthermore, nonanuric patients were significantly higher Zn, Se, and Mo losses than healthy subjects, and anuric patients had a lower excretion of arsenic than healthy subjects.

**Figure 2:**
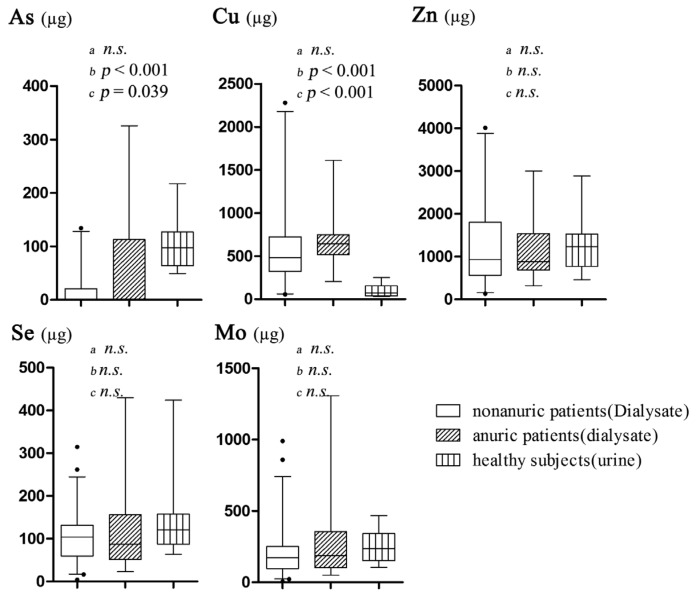
Comparisons of Cu, Zn, Se, Mo, and As excretions per day among dialysate of nonanuric patients and anuric patients and urine of healthy subjects. ^a^ nonanuric patients vs. anuric patients; ^b^ nonanuric patients vs. healthy subjects; ^c^ anuric patients vs. healthy subjects.

**Figure 3:**
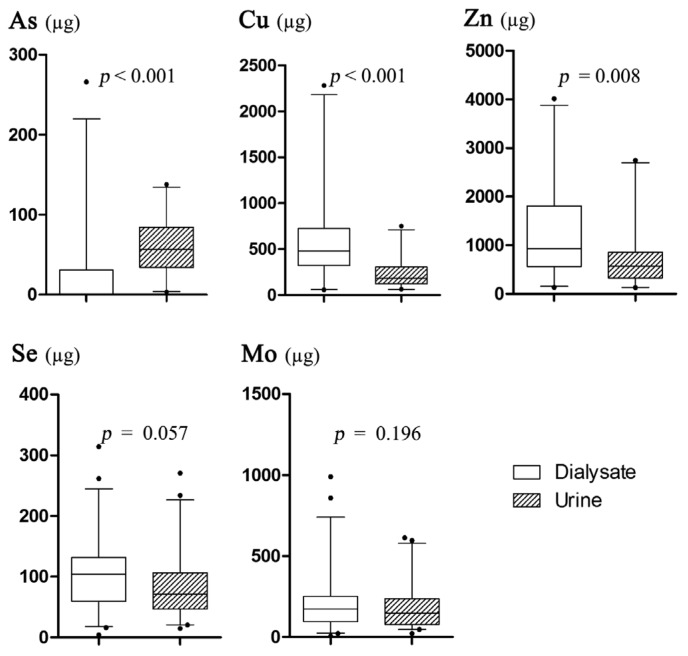
Comparisons of Cu, Zn, Se, Mo, and As excretions per day between dialysate and urine in nonanuric patients.

To analyze the difference, two comparisons were carried out as follows: (1) the comparisons of Cu, Zn, Se, Mo, and As excretions among dialysate of nonanuric and anuric patients and urine of healthy subjects (Figure 2); (2) the comparisons of Cu, Zn, Se, Mo, and As excretions between dialysate and urine in nonanuric patients (Figure 3). There was no significant difference of all these TEs excretion by dialysate between nonanuric and anuric patients. Based on the obtained result, As excretion in dialysate of nonanuric and anuric patients was lower than that in urine of healthy subjects, while the losses of Cu were higher. Besides, it was found that amounts of Cu and Zn excreted in dialysate were higher than those in urine of nonanuric patients, while As excretion was found to be lower.

### 3.3. Correlation Analysis

As presented in Table 3, urinary Cu was associated with urinary Zn, Se, Mo, and As; urinary Zn exhibited moderate correlation coefficients with urinary Mo, Se, and As; urinary Se and Mo were positively related with urinary As. Besides, moderate and positive associations with Cu were observed for Se and Mo in both dialysate and urine. Furthermore, a very strong positive correlation coefficient was noted between Se and Mo in both dialysate and urine. All these associations were significant and positive.

Table 4 shows the correlation coefficient between TE excretions in dialysate and study variables. It showed that Cu excreted by dialysate correlated with creatinine, protein loss, and albumin in dialysate. In addition, both Se and Mo excretion in dialysate were associated positively with creatinine and albumin protein loss in dialysate, while being negatively associated with serum hs-CRP.

In urine (Table 5), Cu was associated with total urinary protein, urinary albumin, urinary creatinine, nPCR, and nPNA. Zn related with serum uric acid, eGFR, nPCR, urinary uric acid, creatinine, and urea nitrogen. Both Se and Mo correlated with eGFR, total urinary protein, urinary albumin, uric acid, creatinine, urea nitrogen, and nPCR.

## 4. Discussion

This cross-sectional study described the daily TEs profile excreted by dialysate and urine in CAPD patients and further assessed the difference of TEs excretions between nonanuric and anuric patients. Interestingly, a close relationship between Se and Mo excretion was also observed.

### 4.1. Characteristics of Daily Trace Element Excretions

In our study, significant excretions of Cu, Zn, Se, Mo, and As occurred through the peritoneal membrane. Similar results for Cu, Zn, and Se were reported in previous studies [32,33]. Our data showed that CKD patients on peritoneal dialysis therapy had a higher excretion of Cu relative to healthy subject. However, studies investigating Cu in CKD patients including patients on PD were inconsistent. Some reports showed normal Cu levels in serum or plasma [11,32,34,35,36], and while others indicated a decreased serum Cu level in CKD patients on dialysis [35,36,37,38]. Guo et al. found that serum Cu levels in PD patients were higher than that in healthy subjects [37,38,39]. Similar inconsistent status also existed in the levels of Zn in CKD patients on dialysis [11,21,32,34,35,36,37,38,39]. The main cause leading to varying results may be that serum or plasma Cu and Zn levels are poorly reflective of actual total body content, which is likely to be attributable to the homeostatic mechanisms of maintaining plasma or serum levels when the intake is excessive or inadequate [40]. The distributions of them also vary in organs [9]. Thus, it is difficult to understand their real status. Investigating the intake or exposure and excretion of them would be a better way of evaluating their deficiency or accumulation.

With regard to Cu, few data were available on intake and excretion of Cu in PD patients, and the intake in HD patients was lower than healthy controls, not reaching 40% of the recommended value [41]. The significantly higher Cu excretion in CAPD patients indicated that Cu deficiency probably occurred, which was thought to worsen anemia in CKD patients [42]. Dietary intake of Zn was lower than dietary reference intakes (DRI) [43]. Given such a huge amount of Zn lost per day, Zn deficiency might also appear in patients on long-term dialysis, especially in nonanuric patients. Zn deficiency can cause or contribute to a number of conditions, including anorexia, dysgeusia, reduced cognitive function, low HDL-cholesterol, and reduced ability to protect against oxidative stress in CKD patients [10]. Based on our analyses, one of main causes for the deficiency of Zn and Cu in CAPD patients was higher losses by dialysate compared with urine.

Although normal Se concentrations have been reported in the advanced stage of CKD [44], lower levels have been noted frequently [37,45,46,47,48], and PD patients were at a higher risk of Se deficiency than HD patients [49]. Despite there being no data available on the daily intake of Se in PD patients, some studies showed it was not meeting the average requirement in general individuals and patients undergoing HD [50,51,52]. Based on these findings, our data further suggested that Se deficiency for CAPD patients, especially in nonanuric patients. Moreover, Se deficiency may contribute to cardiovascular disease, anemia, and immune dysfunction, which are common in patients with renal failure [48,53,54]. Se escaping through peritoneal membrane became another main way of its excretion in CAPD patients based on our study.

There have been few reports on accumulation and deficiency of Mo in CKD patients. The estimation of daily intake of Mo varied between 120 and 240 micrograms per day [55]. On the basis of present information, more than half of patients in this study (the median of daily Mo excretion was 309.49 μg/day, ranged 49.41–1586.19 μg/day) might be deficient in Mo in a long-term CAPD period. Additionally, urinary excretion was the major route of Se and Mo in the general population. Therefore, the daily amounts of Se and Mo lost might lay the foundation for establishing recommendations of TE supplementation for CKD patients undergoing CAPD in further studies.

In agreement with our data on the insufficient excretion of As, Zhang et al. reported that serum As in CAPD patients was significantly higher than in healthy subjects [56]. According to our analysis, arsenic elimination still mainly depended on the kidneys, which further demonstrates the higher risk of arsenic accumulation in anuric patients.

Furthermore, our analysis showed that CAPD patient lost more Cu than healthy subjects regardless of renal excretion of patients, while they discharged lower arsenic. When it was taken into account, although arsenic accumulation was alleviated, the losses of Cu, Zn, Se, and Mo were aggravated in nonanuric patients. That is, nonanuric patients probably suffered from the deficiency of Cu, Zn, Se, and Mo, while anuric patients are at high risk of arsenic accumulation. It is highly consistent with the status of daily excretions of TEs. These differences are clinically important and warrant that nonanuric and anuric patients need to be paid different attentions. In other words, nonanuric patients should take in more Cu, Zn, Se, and Mo by food or supplements, while anuric patients need routine monitoring of the status of arsenic and to prevent from its toxic effect on kidney disease patients.

### 4.2. Association between Se and Mo Excretion

Interestingly, we found that there was a strong association between Se and Mo concentration in both dialysate (β = 0.869, *p* < 0.010) and urine (β = 0.968, *p* < 0.010). A similar relationship was observed in the urine of our healthy subjects (β = 0.927, *p* < 0.010). It is known that Se and Mo are both chemically similar to sulfur (S), and they can form some stable compound [57]. Se deficiency is usually accompanied by Mo deficiency in a natural environment. Additionally, Se and Mo can share the same transporters for sulfate in plants and there are important kind of enzymes containing both Se and Mo in bacteria [58,59]. Furthermore, a Mo-Se cluster was revealed in a well-established cohort study that aimed to identify potential common environmental sources and metabolic pathways using metal-mixtures in urine from participants [60]. From clues mentioned above, we speculated that the potential reason leading to the strong and positive association in our study might be that Se bound with Mo and formed a compound when they were discharged.

In addition, our analysis showed that the clinical variables which were associated with Se excretion were also associated with Mo excretion as well. Some studies reported that acute-phase response, as documented by elevated plasma C-reactive protein levels, causes decreased plasma Se and selenoprotein redistribution [61,62]. It is postulated that a similar change may also exist in Mo distribution, which can be explained by the negative association of hs-CRP with both Se and Mo. Besides, Fang et al. found Mo deficiency might be one of the causes of Keshan Disease which is mainly caused by Se deficiency [63]. Zhang et al. reported a relationship between Mo and Se utilization in prokaryotes [64]. Furthermore, both Se and Mo play a similar role in a recombinant *Escherichia coli* fermentation [65]. Based on the studies mentioned above, our study further indicated a close association between Se and Mo excretion, and the association might even exist in their metabolism and physiological function in human beings. To the best of our knowledge, our results were the first to provide clues for their association in CKD patients. Available data are encouraging and stimulate interest in further studies to clarify the association between Se and Mo in patients with kidney disease and even in the general population.

Finally, we are fully aware of limitations of our study as having small sample size, with data being single-center and cross-sectional. Besides, the number of control individuals may be insufficient. Additional longitudinal investigations with larger numbers of patients and control individuals, especially with respect to the foregoing limitations, are necessary to confirm our results.

## 5. Conclusions

In the present study, it was found that high amounts of essential TEs have been excreted in CAPD patients and TE excretions exceptionally make a distinct profile between nonanuric and anuric patients. Thus, nutritional supplementation is required for those nonanuric patients, while the accumulation of toxic elements like As should be taken into consideration and routinely monitored. In addition, a close relationship between selenium and molybdenum excretion was observed.

## Figures and Tables

**Table 1:** The basic characteristics of CAPD patients.

	Mean ± SD	Median	Minimum	Maximum
Age (years)	43.31 ± 12.67	45.00	19	68
Sex (Male/Female)	32/29			
Body height (cm)	163.14 ± 7.66	163.00	143.00	176.00
Body weight (kg)	57.55 ± 10.06	58.00	36.00	84.70
BMI (kg/m^2^)	21.60 ± 3.20	21.20	14.40	30.80
Duration of uremia (month)	20.03 ± 26.19	14.00	1.00	140.00
Time of dialysis (months)	12.52 ± 13.43	8.00	3.00	56.00
Inflow of dialysate (L)	6.75 ± 1.22	6.00	4.00	8.00
UF (mL/24 h)	215.33 ± 516.32	175.00	−9.00	1600
Ccr (L/w/1.73 (m^2^)	66.90 ± 19.87	64.94	33.31	130.14
Kt/V	2.00 ± 0.41	1.94	1.20	2.80
nPCR	1.10 ± 0.25	1.06	0.67	1.82
nPNA	0.90 ± 0.18	0.88	0.60	1.30
Salb (g/L)	39.41 ± 4.18	39.20	31.30	50.40
hs-CRP (mg/L)	3.33 ± 5.26	1.30	0.10	30.50
FBG (mmol/L)	5.75 ± 1.66	5.35	4.40	15.63
eGFR	5.31 ± 2.20	4.70	2.80	14.80
Total protein lost in dialysate (g/day)	4.89 ± 1.97	4.65	1.58	12.00
Dalb (g/L)	3.58 ± 1.36	3.34	1.08	7.66
Dcre (μmol/L)	680.20 ± 261.31	667.00	4.50	1330.00
Dun (mmol/L)	15.96 ± 4.68	15.84	5.54	28.04
Upro (g/L)	0.60 ± 0.53	0.42	0.01	2.22
Ualb (g/L)	0.36 ± 0.35	0.29	0.01	1.73
UUA (μmol/L)	460.83 ± 254.20	416.50	2.17	1153.00
Ucre (μmol/L)	5234.81 ± 2994.75	4424.50	257.00	12,112.00
Uun (mmol/L)	57.96 ± 33.21	53.00	20.00	202.00

UF: peritoneal ultrafiltration volume; Ccr: weekly creatinine clearance; Kt/V: weekly total urea clearance; nPCR: normalized protein catabolic rate; nPNA: normalized protein nitrogen appearance; Salb: serum albumin; hs-CRP: high-sensitivity C-reactive protein; FGB: fasting blood glucose; eGFR: evaluated glomerular filtration rate; Dpro: Protein loss into the dialysate; Dalb: albumin in dialysate; Dcre: creatinine in dialysate; Dun: urea nitrogen in dialysate; Upro: total urinary protein; Ualb: urinary albumin; UUA: urinary uric acid; Ucre: urinary creatinine; Uun: urinary urea nitrogen; nPNA: normalized protein nitrogen appearance; nPCR: normalized protein catabolic rate.

**Table 2:** Comparison of daily trace element losses between patients and healthy subjects.

	Patients			Healthy Subjects (*n* = 11)
	All (*n* = 61)	Nonanuric Patients (*n* = 45)	Anuric Patients (*n* = 16)	
Age (years)	43.31 ^a^ ± 12.67	43.39 ± 11.11	43.38 ± 16.73	29.82 ± 7.82
Sex (Male/Female)	32/29	20/25	11/5	6/5
BMI (kg/m^2^)	21.60 ± 3.20	21.20 ± 2.99	22.79 ± 3.57	21.06 ± 2.39
Time of dialysis (months)	12.52 (3.00–56.00)	5.00 ^d^ (3.00–51.00)	16.50(3.00–56.00)	-
Cu (μg/day)	672.49 ^a^	771.01 ^b^	653.54 ^c^	72.76
	(205.40–2447.69)	(244.23–2447.69)	(205.40–1610.92)	(29.4–251.17)
Zn (μg/day)	1546.14	1757.65 ^b,d^	1099.30	1232.52
	(321.97–4622.47)	(830.30–4280.55)	(351.97–4622.47)	(463.34–2889.00)
Se (μg/day)	158.76	173.04 ^b,d^	98.19	120.79
	(23.26–548.35)	(94.41–548.35)	(23.26–429.86)	(63.32–423.55)
Mo (μg/day)	309.49	319.45 ^b,d^	219.03	237.20
	(49.41–1586.19)	(143.09–1586.19)	(49.41–1307.71)	(105.16–467.84)
As (μg/day)	69.44	70.67 ^d^	4.8 ^c^	97.81
	(0.01–325.75)	(11.21–169.44)	(0.01–325.75)	(49.61–217.39)

^a^ *p* < 0.050 patients vs. healthy controls; ^b^ *p* < 0.050 nonanuric patients vs. healthy controls; ^c^ *p* < 0.050 anuric patients vs. healthy controls; ^d^ *p* < 0.050 nonanuric patients vs. anuric patients.

**Table 3:** Spearman correlation coefficients between different trace element concentrations excreted in dialysate (β_1_) and in urine (β_2_).

	Cu		Zn		Se		Mo		As	
	β_1_	β_2_	β_1_	β_2_	β_1_	β_2_	β_1_	β_2_	β_1_	β_2_
Cu	1	1								
Zn	0.182	0.458 **	1	1						
Se	0.483 **	0.394 **	−0.011	0.684 **	1	1				
Mo	0.327 *	0.346 *	−0.071	0.607 *	0.869 **	0.968 **	1	1		
As	0.121	0.396 **	0.023	0.510 **	0.027	0.688 **	0.071	0.662 **	1	1

* *p* < 0.050; ** *p* < 0.010.

**Table 4:** Spearman correlation coefficients between study variables and trace element excretions in dialysate per day.

	Cu	Zn	Se	Mo
Salb (g/L)	0.034	0.039	0.201	0.203
hs-CRP (mg/L)	−0.025	−0.012	−0.417 **	−0.431 **
FGB (mmol/L)	0.086	−0.035	−0.074	0.057
eGFR (mL/min/1.73 m^2^)	−0.097	−0.205	−0.022	−0.174
Dpro (g/day)	0.588 **	−0.141	0.394 **	0.314 *
Dalb (g/L)	0.614 **	0.214	0.434 **	0.338 **
Dcre (umol/L)	0.369 **	−0.057	0.333 **	0.347 **
Dun (mmol/L)	−0.083	−0.208	0.061	0.070
nPNA	−0.169	−0.129	−0.021	−0.022
nPCR	−0.243	−0.171	−0.060	−0.088

Salb: serum albumin; FGB: fasting blood glucose; eGFR: evaluated glomerular filtration rate; Dpro: protein lost in dialysate; Dalb: albumin in dialysate; Dcre: creatinine in dialysate; Dun: urea nitrogen in dialysate; nPNA: normalized protein nitrogen appearance; nPCR: normalized protein catabolic rate. * *p* < 0.050; ** *p* < 0.010.

**Table 5:** Spearman correlation coefficients between study variables and urinary trace element excretions per day.

	Cu	Zn	Se	Mo
Salb (g/L)	−0.140	0.105	0.242	0.270
Sua (umol/L)	0.045	0.383 **	0.208	0.249
FGB (mmol/L)	−0.172	0.007	−0.033	−0.019
eGFR	−0.262	0.370 *	0.368 *	0.363 *
Upro (g/L)	0.455 **	−0.212	0.353 *	0.382 **
Ualb (g/L)	0.456 **	0.263	0.374 **	0.413 **
Uua (umol/L)	0.332 *	0.524 **	0.496 **	0.486 **
Ucre (umol/L)	0.014	0.466 **	0.329 *	0.353 *
Uun (mmol/L)	0.236	0.538 **	0.580 **	0.604 **
nPNA	0.320 *	0.046	0.225	0.223
nPCR	0.367 **	0.340 *	0.408 **	0.413 **

Salb: serum albumin; Sua: serum uric acid; FGB: fasting blood glucose; eGFR: evaluated glomerular filtration rate; Upro: total urinary protein; Ualb: urinary albumin; Uua: urinary uric acid; Ucre: urinary creatinine; Uun: urinary urea nitrogen; nPNA: normalized protein nitrogen appearance; nPCR: normalized protein catabolic rate. * *p* < 0.050; ** *p* < 0.010.

## Appendix A

**Table A1:** The limit of quantification and RSD.

	LOQ	LOQ/2	RSD
Cu	0.2336	0.1168	0.8
Zn	0.0010	0.0005	1.0
As	0.0128	0.0064	0.9
Se	0.1237	0.0618	0.8
Mo	0.0036	0.0018	0.7

LOQ: The limits of quantification; RSD: The relative standard deviation.
